# Evaluation and Identification of Promising Introgression Lines Derived From Wild *Cajanus* Species for Broadening the Genetic Base of Cultivated Pigeonpea [*Cajanus cajan* (L.) Millsp.]

**DOI:** 10.3389/fpls.2019.01269

**Published:** 2019-10-22

**Authors:** Shivali Sharma, Pronob J. Paul, C.V. Sameer Kumar, P. Jaganmohan Rao, L. Prashanti, S. Muniswamy, Mamta Sharma

**Affiliations:** ^1^Theme Pre-breeding, International Crops Research Institute for the Semi-Arid Tropics (ICRISAT), Hyderabad, India; ^2 ^Regional Agricultural Research Station, Professor Jayashankar Telangana State Agricultural University, Palem, India; ^3^Regional Agricultural Research Station, Professor Jayashankar Telangana State Agricultural University, Warangal, India; ^4^Regional Agricultural Research Station, Acharya N. G. Ranga Agricultural University, Tirupati, India; ^5^Regional Agricultural Research Station, University of Agricultural Sciences, Kalaburagi, India; ^6^Legume Pathology, International Crops Research Institute for the Semi-Arid Tropics (ICRISAT), Hyderabad, India

**Keywords:** pre-breeding, introgression lines, *Cajanus cajanifolius*, *Cajanus acutifolius*, AMMI, wild *Cajanus species*, pigeonpea

## Abstract

Pigeonpea [*Cajanus cajan* (L.) Millsp.], a multipurpose and nutritious grain legume crop, is cultivated for its protein-rich seeds mainly in South Asia and Eastern and Southern Africa. In spite of large breeding efforts for pigeonpea improvement in India and elsewhere, genetic enhancement is inadequate largely due to its narrow genetic base and crop susceptibility to stresses. Wild *Cajanus* species are novel source of genetic variations for the genetic upgradation of pigeonpea cultivars. In the present study, 75 introgression lines (ILs), derived from crosses involving cultivated pigeonpea variety ICPL 87119 and wild *Cajanus cajanifolius* and *Cajanus acutifolius* from the secondary gene pool, were evaluated for yield and yield-attributing traits in diverse environments across locations and years. Restricted maximum likelihood (REML) analysis revealed large genetic variations for days to 50% flower, days to maturity, plant height, primary branches per plant, pods per plant, pod weight per plant, 100-seed weight, and grain yield per plant. Superior ILs with mid-early to medium maturity duration identified in this study are useful genetic resources for use in pigeonpea breeding. Additive main effects and multiplicative interaction (AMMI) analysis unfolded large influence of environment and genotype × environment interaction for variations in yield. A few lines such as ICPL 15023 and ICPL 15072 with yield stability were identified, while a number of lines were completely resistant (0%) to sterility mosaic diseases and/or *Fusarium* wilt. These lines are novel genetic resources for broadening the genetic base of pigeonpea and bring yield stability and stress tolerance. High-yielding lines ICPL 15010, ICPL 15062, and ICPL 15072 have been included in the initial varietal trials (IVTs) of the All India Coordinated Research Project (AICRP) on pigeonpea for wider evaluation across different agro-ecological zones in India for possible release as variety(ies).

## Introduction

Pigeonpea [*Cajanus cajan* (L.) Millsp.], originating in India, is the sixth most important grain legume crop of the tropics and subtropics and grown for multiple uses. It is an often-cross-pollinated diploid (2*n* = 2*x* = 22) crop. Globally, 6.81m t of pigeonpea grains was produced from 7.02m ha with an average productivity of 0.97 t ha^−1^ (FAOSTAT, 2017). Although its presence has been noted in many countries, India and Myanmar in South Asia and Kenya, Tanzania, Malawi, Uganda, and Mozambique in Eastern and Southern Africa are the major pigeonpea-producing countries (FAOSTAT, 2017). India contributed about 72% of global pigeonpea production. Disproportionate yield gaps were noted between potential (2.5–3.0 t ha^−1^) and average (∼0.9 t ha^−1^) yields in India (Bhatia et al., 2006). The average yield in India remained around 0.9 t ha^−1^ for the past six decades (FAOSTAT, 2017). This yield gap is mainly due to the exposure of the crop to biotic stresses such as *Fusarium* wilt (FW; caused by *Fusarium udum* Butler), sterility mosaic diseases (SMD; caused by pigeonpea sterility mosaic virus transmitted by eriophyid mite, *Aceria cajani* Channabasavanna), phytophthora blight (*Phytophthora drechsleri* Tucker f. sp. *cajani*), pod borer (*Helicoverpa* sp.), and pod fly (*Melanagromyza obtusa*) and abiotic stresses such as waterlogging, salinity, and frost/cold as well as due to its cultivation in marginal environments with limited inputs (Sharma and Upadhyaya, 2016).

Like other legumes, domestication bottlenecks also contributed to the narrow genetic base in Pigeonpea (Kassa et al., 2012). Breeders often use their own working collection consisting of elite breeding and some germplasm lines as parents in crossing. This results in recirculating the same germplasm, leading to the narrow genetic base of the released cultivars. In pigeonpea, T-1 and T-90 were the most frequently used germplasm as parents in breeding programs in India (Kumar et al., 2004). The polymorphic survey of a set of *Cajanus* accessions has also indicated the lack of genetic diversity within the cultivated gene pool (Kumar et al., 2018). Furthermore, the natural defense mechanism in improved cultivars has been lost during intense selection for high yield, which may result in the genetic vulnerability of crop cultivars to a number of biotic and abiotic stresses (Tanksley and McCouch, 1997). Overall, the narrow genetic base of pigeonpea cultivars and lack of high levels of resistance/tolerance to important biotic and abiotic stresses in cultivated gene pool and/or breeder working collection hinders its genetic improvement and results in low genetic gains.

Wild *Cajanus* species are the reservoir of many useful genes and hold great potential for pigeonpea improvement. The ICRISAT genebank has the global responsibility of collecting, conserving, and distributing pigeonpea germplasm comprising landraces, modern cultivars, genetic stocks, mutants, and wild *Cajanus* species. It holds over 13,200 accessions of cultivated pigeonpea and 555 accessions belonging to 66 species of six genera in genus *Cajanus* from 74 countries (Upadhyaya et al., 2013). This germplasm collection based on the crossability relationship between cultivated and wild pigeonpea has been grouped into three gene pools (Sharma, 2017) (**Table 1**).

**Table 1:** Pigeonpea gene pool classification.

Gene pool	Taxon	Features
Primary gene pool	*Cajanus cajan* (L.) Millsp. (all cultigens)	Cross-compatible among themselves
Secondary gene pool	*Cajanus acutifolius* (F.Muell.) Maesen, *Cajanus albicans* (Wight & Arn.) Maesen, *Cajanus cajanifolius* (Haines) Maesen, *Cajanus cinereus* (F.Muell.), *Cajanus confertiflorus* (F.Muell.), *Cajanus lanceolatus* (W. Fitzg.) Maesen, *Cajanus latisepalus* Maesen, *Cajanus lineatus* (Wight & Arn.) Maesen, *Cajanus reticulatus* (Dryand.) F.Muell., *Cajanus scarabaeoides* (L.) Thouars, *Cajanus sericeus* (Baker) Maesen, *Cajanus trinervius* (DC.) Maesen	Cross-compatible with cultivated pigeonpea
Tertiary gene pool	*Cajanus crassus* (King) Maesen, *Cajanus goensis* Dalzell, *Cajanus mollis* (Benth.) Maesen, *Cajanus platycarpus* (Benth.) Maesen, *Cajanus rugosus* (Wight & Arn.) Maesen, *Cajanus heynei, Cajanus kerstingii, Cajanus volubilis,* and other Cajaninae such as *Rhynchosia* Lour., *Dunbaria* W. and A., and *Eriosema* (DC.) Reichenb	Cross-incompatible with cultivated pigeonpea

Multiple sources of resistance/tolerance to stress have been reported among wild *Cajanus* species—SMD (Kulkarni et al., 2003; Rao et al., 2003); phytophthora blight (Rao et al., 2003); alternaria blight (*Alternaria* *tenuissima*; Sharma et al., 1987); pod borer (*Helicoverpa armigera*) (Rao et al., 2003; Sujana et al., 2008; Sharma et al., 2009); pod fly (Saxena et al., 1990; Rao et al., 2003); root-knot nematodes (*Meloidogyne* spp.; Sharma et al., 1993a; Sharma et al., 1993b; Sharma et al., 1994; Rao et al., 2003); salinity (Subbarao, 1988; Subbarao et al., 1991; Rao et al., 2003; Srivastava et al., 2006); and drought (Rao et al., 2003). Pigeonpea, by nature, is a photosensitive crop. A few wild *Cajanus* accessions, however, were reported as insensitive to photoperiod (Rao et al., 2003).

Cultivated pigeonpea is believed to originate in India (Vavilov, 1951; van der Maesen, 1980). In this study, two wild *Cajanus* species from a secondary gene pool, *Cajanus acutifolius* and *Cajanus cajanifolius*, belonging to different geographic origins were crossed with a popular pigeonpea variety, ICPL 87119 (also known as ‘Asha’), to generate interspecific populations following advanced backcross approach. *C. acutifolius* accession ICPW 12 (syn. ICP 15613) is a native of Australia and reported to be resistant to *H. armigera* (Sujana et al., 2008), whereas *C. cajanifolius* accession ICPW 29 (syn. ICP 15630) is of Indian origin and the progenitor of cultivated pigeonpea (van der Maesen, 1980). The main aim of this investigation was to (a) create new genetic variability with minimum linkage drag by utilizing two wild *Cajanus *species of different geographic origins as donors and popular pigeonpea cultivars as recipients following advanced backcross approach and (b) identify promising introgression lines (ILs) having good agronomic performance and disease resistance for ready use in pigeonpea breeding programs. These promising ILs will enrich variability in the primary gene pool, and their utilization in breeding programs will assist in developing new climate-resilient cultivars with a broad genetic base, which in turn will enhance the genetic gains in pigeonpea.

## Materials and Methods

### Development of Pre-Breeding Populations

Using two wild *Cajanus* accessions, ICPW 12 (*C. acutifolius*) and ICPW 29 (*C. cajanifolius*), natives of Australia and India, respectively, and popular pigeonpea cultivar ICPL 87119, two pre-breeding populations were developed at ICRISAT, Patancheru, India. ICPL 87119 (Asha) is a medium-duration leading variety widely cultivated in India (Jain et al., 1995) while ICPW 12 and ICPW 29 were reported to have high levels of resistance against pod borer (Sujana et al., 2008).

ICPL 87119 was used as the female parent, whereas wild species accessions were used as the male parent to generate F_1_ hybrids. In each cross, true F_1_s were selected based on leaves, flowers, and pod morphology and subsequently backcrossed with ICPL 87119 to produce BC_1_F_1_ seeds. Similarly, true BC_1_F_1_ plants in both crosses were identified based on morphological traits, and the confirmed BC_1_F_1_ plants were used for the second backcross with ICPL 87119 to produce BC_2_F_1_ seeds. True BC_2_F_1_ plants were selfed twice to produce BC_2_F_3_ populations that were subsequently advanced to produce stable ILs, 149 in ICPL 87119 × ICPW 12 (designated as Pop I) and 183 in ICPL 87119 × ICPW 29 (designated as Pop II). Considerable variability for plant type and morpho-agronomic traits was observed between and within lines in both populations. In the first round of selection, stable lines with no segregation but having a good agronomic performance and differing in maturity such as mid-early (140–180 days) to medium (161–180 days to maturity) maturity, high seed yield, and 100-seed weight were selected. Overall, 30 stable ILs (12 ILs from Pop I and 18 ILs from Pop II) were selected to assess their agronomic performance across four locations during the 2016 rainy season in India (**Table 2**).

**Table 2:** Details of experiments conducted across locations/years.

Experimental details		MET 01	MET 02	Trial 03	Trial 04
Genotypes	Number of genotypes	15 ILs + 2 checks	15 ILs + 2 checks	16 ILs + 3 checks	29 ILs + 3 checks
	Genotype identity	ICPL # 15006, 15007, 15010, 15017, 15019, 15023, 15041, 15042, 15057, 15060, 15062, 15065, 15071, 15075, 15085	ICPL # 15003, 15004, 15014, 15021, 15024, 15030, 15034, 15040, 15046, 15054, 15058, 15067, 15072, 15077, 15079	ICPIL # 17148, 17149, 17150, 17151, 17152, 17153, 17154,17155,17156,17157,17158,17159,17160,17161,17162,17163	ICPIL # 17164, 17165, 17166, 17167, 17168, 17169, 17170, 17171,17172,17173,17174, 17175, 17176, 17177, 17178, 17179, 17180, 17181,17182, 17183, 17184, 17185, 17186, 17187, 17188, 17189,17190, 17191,17192
	Checks	ICPL 87119 and ICP 8863	ICPL 87119 and ICP 8863	ICPL 85010, ICPL 87119, and ICP 8863	ICPL 85010, ICPL 87119, and ICP 8863
Environments	Number of locations	4	4	1	1
	Name of locations	Patancheru, Warangal, Tirupati, Gulbarga	Patancheru, Warangal, Tirupati, Gulbarga	Patancheru	Patancheru
	Number of seasons	One (2016 rainy season)	One (2016 rainy season)	Two (2016 rainy and 2017 rainy seasons)	Two (2016 rainy and 2017 rainy seasons)

The second round of selection was made to exploit within-line variability in the remaining lines in both populations. The selection was made in two stages. At the first stage, almost stable lines showing some segregation and overall good agronomic performance were selected. At the second stage, single plants were selected based on the visual observations and overall plant aspect score from each of the selected lines in both populations. Overall, 16 single plants from 16 selected lines in Pop I and 29 single plants from 29 selected lines in Pop II were selected for evaluation over years at ICRISAT, Patancheru (**Table 2**).

### Evaluation of Promising ILs for Yield-Related Traits

For precise phenotyping with minimum microenvironment errors across locations, two multilocation evaluation trials (designated as “MET”) were constituted using 30 stable ILs. For this, 30 ILs were randomly divided into two sets: set I with 15 ILs (five from Pop I and 10 from Pop II) was evaluated in MET 01, and set II with the remaining 15 ILs (seven ILs from Pop I and eight ILs from Pop II) was evaluated in MET 02. Both MET 01 and MET 02 trials were conducted under rainfed conditions across four locations, Patancheru, Kalaburagi, Tirupati, and Warangal, during the 2016 rainy season. These locations were selected based on the high importance of pigeonpea crop in these areas, especially under rainfed conditions (**Table S1**). Both MET 01 and MET 02 were conducted in “Vertisols” at Patancheru, Kalaburagi, and Warangal and in “Alfisols” at Tirupati. Two popular pigeonpea varieties [ICPL 87119, (Jain et al., 1995) and ICP 8863, also known as ‘Maruti’, (ICRISAT, 1993)] were used as checks in each trial.

Using 16 single plant selections (SPSs) from Pop I and 29 SPSs from Pop II, two trials, designated as “Trial 03” and “Trial 04,” respectively, were conducted at ICRISAT, Patancheru, for the evaluation of yield-related traits during the 2016 and 2017 rainy seasons. In both Trial 03 and Trial 04, three checks, ICPL 87119, ICP 8863, and ICP 85010, were included in the evaluation studies.

Each trial across all locations/seasons was conducted in a randomized block design with three replications. Plot size was a four-row plot of 4-m length with 1.2-m row-to-row spacing in the MET 01 and MET 02 and a four-row plot of 4-m length with 75-cm spacing in Trial 03 and Trial 04. Manual weeding and spraying of insecticide were done to control weeds and insect-pest damage. All other recommended agronomic practices were followed for raising a healthy crop.

Data were recorded on days to 50% flower, days to maturity, plant height (cm), 100-seed weight (g), and grain yield per plant (g) in MET 01 and MET 02 at each location. In Trial 03 and Trial 04, data were recorded on days to first flower, days to 50% flower, plant height (cm), primary branches per plant, pods per plant, pod weight per plant (g), 100-seed weight (g), and grain yield per plant (g). Data on days to first flower, days to 50% flower, and days to maturity were recorded on a plot basis, whereas plant height, primary branches, pods per plant, pod weight per plant, 100-seed weight, and grain yield per plant were recorded on five randomly selected representative plants per plot following pigeonpea descriptors (IBPGR and ICRISAT, 1993).

### Screening for FW and SMD Resistance

A total of 45 ILs (16 ILs from Trial 03 and 29 ILs from Trial 04) were screened for FW and SMD in the sick plot under artificial epiphytotic conditions during the 2017 rainy season at ICRISAT, Patancheru, and 32 promising resistant ILs (12 ILs from Trial 03 and 20 ILs from Trial 04) were further evaluated during the 2018 rainy season for confirming resistance. For FW screening, chopped wilted pigeonpea was incorporated in the sick plot to maintain a threshold level of the *F. udum*, the wilt pathogen. ICP 2376, a highly wilt-susceptible cultivar, was planted after every five rows to serve as an indicator/infector row. For SMD screening, SMD-infested leaves (Patancheru isolate) were inoculated in every plant of the ILs at a two-leaf stage following the leaf staple technique (Nene et al., 1981). To provide a good source of virus inoculum, a highly susceptible cultivar, ICP 8863, was planted one month in advance of the regular planting after every five rows of test entries to serve as an indicator/infector row. Special care was taken during planting of test ILs and susceptible cultivar in the wind direction to facilitate the virus transmission through mites. The percent disease incidence was calculated using the formula: Percent disease incidence = (no. of plants infected in a row/total no. of plants in a row) × 100. Based on the disease incidence, ILs were categorized as resistant (0–10% diseases incidence), moderately resistant (10.1–20%), moderately susceptible (20.1–40%), and susceptible (>40%) (Pande et al., 2012).

### Statistical Analysis

Replicate-wise data on five agronomic traits in MET 01 and MET 02 and eight agronomic traits in Trial 03 and Trial 04 were analyzed using restricted maximum likelihood (REML) methods for each location considering genotypes as a random effect and replications as a fixed effect in the mixed-model procedure (Patterson and Thompson, 1971). Variance components due to genotypes $\left(\sigma_{\text{g}}^{2}\right)$ and their standard errors were determined. Environment-wise best linear unbiased predictors (BLUPs) were calculated for each genotype in each trial. The significance of variance components was tested using respective standard errors. Heritability (*H* ^2^, broad sense) at an individual environment was estimated from the following formula:

$$H^{2}=\frac{\sigma_{\text{g}}^{2}}{\sigma_{\text{g}}^{2}+\sigma_{\text{e}}^{2}/r}$$

where $\sigma_{\text{g}}^{2}$ is the variance component due to genotypes, $\sigma_{\text{e}}^{2}$ is the variance component due to error, and *r* is the number of replications.

A phenotypic distance matrix was created by calculating the differences between each pair of entries for each trait. The diversity index was calculated by averaging the differences in the phenotypic values for each trait divided by the respective range (Johns et al., 1997). The mean diversity, minimum diversity, and maximum diversity were calculated, and the accessions showing the minimum diversity and maximum diversity were identified in each trial.

To study the adaptability and yield stability of the ILs across different locations, additive main effects and multiplicative interaction (AMMI) analysis was performed (Gauch, 1992). The basic model for AMMI is based on the additive variance from the multiplicative variance and the principal component analysis (PCA) as detailed here:

$$Y_{ij}=µ+g_{i}+e_{i}+\overset{N}{\underset{n=1}{\sum }}\tau_{n\;}\Upsilon_{in}\;\delta_{jn}+\varepsilon_{ij}$$

where *Y* *_ij_* is the yield of the *i* ^th^ genotype (*i* = 1, …, *L*) in the *j* ^th^ environment (*j* = 1, …, *J*); µ is the grand mean; *g* *_i_* and *e* *_j_* are the genotype and environment deviations from the grand mean, respectively; τ*_n_* is the eigenvalue of the PCA axis *n*; γ*_in_* and δ*_jn_* are the genotype and environment principal component (PC) scores for axis *n*; *N* is the number of PCs retained in the model; and ε*_ij_* is the error term.

AMMI stability value (ASV) was calculated for each IL according to the relative contribution of the PC axis scores (IPCA1 and IPCA2) to the interaction sum of squares (SS).

The ASV was estimated as described by Purchase et al. (2000):

$$ASV=\sqrt{{\left[\frac{IPCA1_{\text{Sum of squares}}}{IPCA2_{\text{Sum of squres}}}\left(IPCA1_{\text{score}}\right)\right]}^{2}}\;+{\left(IPCA2_{\text{score}}\right)}^{2}\;$$

where *IPCA*1_Sum of squares_/*IPCA*2_Sum of squares_ is the weight derived from dividing the IPCA1 SS [from the AMMI analysis of variance (ANOVA) table] by the IPCA2 SS. The larger the IPCA score is, either negative or positive, the more adapted a genotype is to a certain environment. Conversely, smaller ASV scores indicate a more stable genotype across environments.

Genotype selection index (GSI) was estimated (Farshadfar, 2008) using the sum of the ranking based on yield and ranking based on the ASV as

GSI=RASV+RY

where *RASV* is the rank of the genotypes based on the ASV and *RY* is the rank of the genotypes based on yield across environments.

All analyses were performed in Genstat 19 (VSN International, Hemel Hempstead, UK, web page: genstat.co.uk).

## Results

### Variance Components, Trait Variability, and Heritability

REML analysis showed that variances due to genotypes (σ^2^g) were significant for days to 50% flower, days to maturity, plant height, 100-seed weight, and grain yield per plant across four locations in both MET 01 and MET 02, indicating the presence of significant variability among genotypes (**Table 3**). In Trial 03 (**Table 4**) and Trial 04 (**Table 5**) also, significant variability was observed among genotypes for days to first flower, days to 50% flower, plant height, primary branches per plant, pods per plant, 100-seed weight, pod weight per plant, and grain yield per plant in 2016 and 2017 at ICRISAT, Patancheru.

**Table 3:** Variance components due to genotypes $\left(\sigma_{\text{g}}^{2}\right)$, mean, range, and heritability (*H* ^2^) of agronomic traits across locations of MET 01 and MET 02 during the 2016 rainy season.

Statistical parameter	Location	MET 01					MET 02				
		DF50	DM	PH	HSW	GYPP	DF50	DM	PH	HSW	GYPP
Genotypic variance	Patancheru	12.6*	20.3*	131.4*	0.8*	29.9*	15.8*	19.2*	76.3*	0.4*	52.3*
	Kalaburagi	28.1*	16.5*	119.6*	1.1*	142.8*	10.4*	14.1*	36.9*	0.7*	60.4*
	Tirupati	29.2*	43.7*	105.4*	0.6*	77.0*	56.4*	63.1*	34.7*	0.2*	93.9*
	Warangal	4.6*	8.6*	214.4*	1.3*	67.0*	3.2*	2.4*	132.6*	0.8**	63.1*
Mean^†^	Patancheru	106.0^a^	147.0^a^	104.8^a^	10.8^a^	41.7^a^	105.0^a^	146.0^a^	96.09^a^	10.0^ab^	43.5^a^
	Kalaburagi	109.0^b^	157.0^b^	145.6^c^	10.4^ab^	68.3^c^	106.0^a^	153.0^b^	142.1^b^	9.7^a^	59.6^c^
	Tirupati	110.0^b^	163.0^c^	135.9^b^	10.3^ab^	48.9^b^	112.0^b^	161.0^c^	131.0^c^	10.6^b^	50.5^b^
	Warangal	125.0^c^	169.0^d^	151.6^c^	9.7^b^	41.8^a^	124.0^c^	170.0^d^	155.2^a^	10.0^ab^	42.0^a^
Range	Patancheru	104–109	143–153	80.0–129.0	8.4–12.5	34.3–54.0	101–113	141–153	80.0–105.0	9.2–11.9	27.2–55.0
	Kalaburagi	104–119	151–164	130.5–169.5	9.0–13.5	51.7–93.1	100–112	146–160	131.0–153.0	8.1–11.5	46.5–74.4
	Tirupati	104–119	151–170	120.6–153.8	9.6–12.9	36.4–63.8	103–125	143–170	123–141.8	9.9–11.3	37.7–62.8
	Warangal	121–127	164–176	118.7–173.5	8.2–13.1	28.1–55.6	121–126	168–171	141.2–173.4	8.6–11.4	27.8–53.6
Heritability (*H* ^2^)	Patancheru	93.4	90.1	90.8	93.5	88.9	89.2	90.6	89.9	89.4	90.3
	Kalaburagi	99.8	91.4	91.0	99.0	94.4	98.1	98.8	87.6	99.7	91.4
	Tirupati	89.4	81.4	92.2	87.0	75.3	98.0	88.2	72.4	82.6	80.2
	Warangal	83.7	90.7	82.8	98.8	85.8	77.0	80.4	75.2	96.9	90.6

^#^DF50, days to 50% flower; DM, days to maturity; PH, plant height; HSW, 100-seed weight; GYPP, grain yield per plant.

*Significant at P ≤ 0.05. ^†^Mean values were tested using Newman–Keuls test, and means with different alphabets are significantly different at P ≤ 0.05.

**Table 4:** Variance components due to genotypes $\left(\sigma_{\text{g}}^{2}\right)$, mean, range, and heritability (*H* ^2^) of agronomic traits in Trial 03 at ICRISAT Patancheru during the 2016 and 2017 rainy seasons.

Statistical parameter	Genotypic variance		Mean^†^		Range		Heritability (*H* ^2^)	
Traits	2016	2017	2016	2017	2016	2017	2016	2017
**DF** **^#^**	22.4*	197.3*	98.0^a^	120.0^b^	95–103	116–127	92.6	99.2
**DF50**	42.1*	219.8*	106.0^a^	128.0^b^	102–111	123–134	95.9	99.4
**PH**	49.5*	1,208.9*	149.5^a^	254.3^b^	137.2–165.5	236.4–267	78.2	99.1
**NPB**	12.3*	10.1*	30.4^a^	22.8^b^	26–34	20–28	74.1	80.5
**PPP**	1,733.0*	762.0*	190.7^a^	178.8^b^	98–269	128–220	80.0	65.6
**PWPP**	347.8*	181.7*	60.0^a^	65.8^a^	31.8–104.9	41–82.2	86.9	85.3
**HSW**	0.4*	0.8*	10.9^a^	10.5^a^	9.6–12	8.7–11.5	76.4	93.5
**GYPP**	90.7*	98.9*	30.6^a^	44.5^b^	15.1–48.4	24.8–57.3	89.4	85.8

^#^DF, days to first flower; DF50, days to 50% flower; PH, plant height; NPB, number of primary branches; PPP, pods per plant; PWPP, pod weight per plant; HSW, 100-seed weight; GYPP, grain yield per plant.

*Significant at P ≤ 0.05. ^†^Mean values were tested using Newman–Keuls test, and means with different alphabets are significantly different at P ≤ 0.05.

**Table 5:** Variance components due to genotypes $\left(\sigma_{\text{g}}^{2}\right)$, mean, range, and heritability (*H* ^2^) of agronomic traits in Trial 04 at ICRISAT Patancheru during the 2016 and 2017 rainy seasons.

Statistical parameter	Genotypic variance		Mean^†^		Range		Heritability (*H* ^2^)	
Traits	2016	2017	2016	2017	2016	2017	2016	2017
DF^#^	19.1*	122.4*	98.0^a^	120.0^b^	94–104	118–125	87.9	99.8
DF50	20.4*	140.4*	104.0^a^	128.0^b^	100–109	125–134	89.8	99.8
PH	102.0*	189.1*	162.7^a^	187.9^b^	145.6–188.9	182.3–195.9	83.1	97.9
NPB	14.8*	14.4*	31.6^a^	29.7^b^	27–36	25–35	73.2	78.4
PPP	888.0*	2,622.2*	206.2^a^	197.2^a^	165.9–252	158–425.1	61.4	89.7
PWPP	150.6*	542.2*	69.1^a^	76.4^a^	52.2–88.7	59.4–182.5	76.6	92.7
HSW	0.6*	0.7*	10.9^a^	10.8^a^	9.6–12.1	9.1–12.1	70.7	91.9
GYPP	65.8*	95.9*	40.0^a^	51.0^b^	28.8–54.4	39.1–76.1	77.4	82.0

^#^DF, days to first flower; DF50, days to 50% flower; PH, plant height, NPB, number of primary branches; PPP, pods per plant; PWPP, pod weight per plant; HSW, 100-seed weight; GYPP, grain yield per plant.

^*^Significant at P ≤ 0.05. ^†^Mean values were tested using Neman–Keuls test, and means with different alphabets are significantly different at P ≤ 0.05.

Large variation in range and means were noted in individuals as well across locations (**Table 3**). The Newman–Keuls test of significance for mean values showed significant differences in the performance of genotypes across four locations for most of the traits in both MET 01 and MET 02. ILs in MET 01 flowered and matured significantly earlier at Patancheru, were taller at Kalaburagi and Warangal, but produced maximum grain yield at Kalaburagi (**Table 3**). In MET 02 also, ILs flowered and matured early at Patancheru, were significantly taller at Warangal, and produced higher grain yield at Kalaburagi (**Table 3**).

Significant differences in mean performance were also noted for most traits in Trial 03 and 04 at Patancheru. In both trials, the ILs flowered early in 2016, were taller in 2017, had more primary branches and pods per plant in 2016, but had higher grain yield in 2017 (**Tables 4** and **5**).

High heritability (*H* ^2^) (>70%) was recorded for most of the traits in MET 01 and MET 02 (**Table 3**) as well as in Trial 03 (**Table 4**) and Trial 04 (**Table 5**).

### Phenotypic Diversity and Identification of Promising High-Yielding ILs

The mean phenotypic diversity index across four locations varied from 0.125 (Patancheru) to 0.149 (Tirupati) in MET 01 and from 0.138 (Kalaburagi) to 0.185 (Warangal) in MET 02. In Trial 03, the mean phenotypic diversity index was 0.1059 in 2016 and 0.1143 in 2017, and in Trial 04, it was 0.1164 in 2016 and 0.0.0637 in 2017. The maximum diversity was observed between ICPL 15065 and ICP 8863 at Patancheru and Kalaburagi, between ICPL 15060 and ICPL 15007 at Warangal, and between ICPL 87119 and ICPL 15006 at Tirupati in MET 01 (**Table S2a**). Similarly, in MET 02, the maximum diversity was observed between ICP 8863 and ICPL 15040 at Patancheru and ICP 8863 and ICPL 15079 at Kalaburagi (**Table S2a**). Lines showing maximum diversity were also identified in Trial 03 and Trial 04 (**Table S2b**).

ICPL 15065 was the most diverse accession across the three locations, Patancheru, Kalaburagi, and Tirupati, whereas ICPL 15010 was similar to ICPL 87119 across most locations in MET 01 (**Tables S2a** and **S2c**). In MET 02, ICPL 15014 was the most diverse accession across two locations (Patancheru and Warangal). Similarly, ICPIL 17155 and ICPIL 17156 were the most diverse accessions in Trial 03, and ICPIL 17167 was the most diverse in Trial 04 in 2016 (**Table S2d** **)**. Three lines with maximum diversity–similarity with ICPL 87119 were also identified (**Table S3**).

ILs, in general, flowered early or at par with the popular high-yielding pigeonpea variety ICPL 87119 (Asha) across all four trials. Promising high-yielding ILs were identified (**Table 6**). Most of the ILs in MET 01 across four locations yielded at par with ICPL 87119. However, nine lines at Kalaburagi (20% to 62% yield superiority over ICPL 87119) and two each at Tirupati (65% and 69% yield superiority) and Warangal (25% and 37% yield superiority) were significantly higher yielding than ICPL 87119. Of these, six ILs at Kalaburagi, one IL at Tirupati, and one at Warangal also matured significantly earlier than ICPL 87119 and had a 100-seed weight ranging from 9.5 to 10.5 g (**Table S4a**). ICPL 15085 yielded a significantly higher grain yield at Kalaburagi (over 20% yield superiority), Tirupati (over 65% yield superiority), and Warangal (over 25% yield superiority) and was similar to ICPL 87119 at Patancheru. This IL had a 100-seed weight ranging from 9.0 to 10.7 g across four locations (**Table S4a**). ICPL 15019 was found to be significantly higher yielding at Warangal (∼37% yield superiority) and Kalaburagi (over 35% yield superiority). Similarly, ICPL 15062 exhibited significantly higher grain yield than ICPL 87119 at Kalaburagi (∼30% yield superiority) and Tirupati (over 45% yield superiority). ICPL 15065 combined high grain yield and the highest 100-seed weight (12.5 to 13.5 g) across four locations (**Table S4a**).

**Table 6:** Promising high-yielding introgression lines identified across locations in MET 01 and MET 02 during the 2016 rainy season.

Location	MET 01		MET 02	
	Check	Superior or similar to ICPL 87119	Check	Superior or similar to ICPL 87119
Patancheru	ICPL 87119 (48.2 g)	ICPL # 15065, 15085,15006, 15010, 15041, 15065, 15071, 15075, 15085	ICPL 87119 (38.1 g)	ICPL # **15003**, **15014**, **15021**, **15030**, **15046**, **15058**, **15072**, **15077**, 15067, 15079,15004, 15024, 15034, 15040, 15054
Kalaburagi	ICPL 87119 (57.6 g)	ICPL # **15017**, **15019**, **15023**, **15041**, **15042**, **15062**, **15071**, **15075**, **15085**, 15006, 15007, 15010, 15065, 15057, 15060	ICPL 87119 (43.5 g)	ICPL # **15003**, **15004**, **15014**, **15021**, **15024**, **15030**, **15034**, **15040**, **15046**, **15058**, **15067**, **15072**, **15077**, **15079**, 15054
Tirupati	ICPL 87119 (47.2 g)	ICPL # **15062**, **15085**, 15006, 15010, 15019, 15023, 15042, 15060, 15075, 15017, 15041, 15057, 15065, 15071	ICPL 87119 (37.8 g)	ICPL # **15004**, **15014**, **15021**, **15030**, **15040**, **15054**, **15072**, **15077**, 15024, 15046, 15058, 15067, 15079, 15003, 15034
Warangal	ICPL 87119 (42.5 g)	ICPL # **15019**, **15085**, 15007, 15017, 15023, 15065, 15071, 15006, 15010, 15057, 15060, 15062	ICPL 87119 (41.6 g)	ICPL # **15004**, **15067**, **15072**, 15014, 15024, 15046, 15077, 15079, 15003, 15021, 15030, 15040

Bold emphasis indicates significantly better lines at the 0.5% level of significance.

In MET 02, 14 ILs at Kalaburagi (∼22–71% yield superiority), eight each at Patancheru (∼19–45% yield superiority) and Tirupati (∼41–75% yield superiority), and three at Warangal (∼21–32% yield superiority) significantly out-yielded ICPL 87119 (**Table 6**). On an average, ICPL 15072 across four locations and ICPL 15077, ICPL 15014, ICPL 15021, and ICPL 15030 across three locations (Patancheru, Kalaburagi, and Tirupati) out-yielded ICPL 87119 by ∼50% and 45% (**Table S4b**).

In Trial 03, the grain yield of most of the ILs was similar to that of ICPL 87119 (**Tables 7** and **S5a**). In Trial 04, nine ILs in the 2016 rainy season and only one IL, ICPL 17149, were significantly better than ICPL 87119 for grain yield per plant (**Table 7** and **S5b**). Overall, six ILs (ICPIL # 17165, 17167, 17168, 17169, 17178, and 17188) produced more pods and higher pod weight than ICPL 87119. Based on consistent performance in 2016 and 2017, ICPIL 17165 and ICPIL 17167 were found promising for higher grain yield, pod numbers, and pod weight and days to 50% flower at par with ICPL 87119 (**Table S5b**).

**Table 7:** Promising high-yielding lines identified in Trial 03 and Trial 04 during the 2016 and 2017 rainy seasons at ICRISAT, Patancheru.

	Trial 03		Trial 04	
	Check	Superior or similar to ICPL 87119	Check	Superior or similar to ICPL 87119
Rainy 2016	ICPL 87119 (39.7 g)	ICPIL # 17148, 17151, 17152, 17153, 17157, 17158, 17160, 17161, 17162, 17163	ICPL 87119 (30.9 g)	ICPIL # 17164, 17165, 17166, 17167, 17168,17169, 17170, 17171, 17172, 17173, 17174, 17175, 17176, 17177, 17178, 17179, 17180, 17181, 17182, 17183, 17184, 17185, 17186, 17187, 17188, 17189, 17190, 17191, 17192
Rainy 2017	ICPL 87119 (53.4 g)	ICPIL # 17148, 17150, 17151, 17152, 17153, 17154, 17157, 17158, 17159, 17160, 17162	ICPL 87119 (51.4 g)	ICPIL # 17164, 17165, 17166, 17167, 17168, 17169, 17170, 17171, 17172, 17173, 17174, 17175, 17176, 17177, 17178, 17179, 17180, 17181, 17182, 17183, 17184, 17185, 17186, 17187, 17188, 17189, 17190, 17191, 17192

Bold emphasis indicates significantly better lines at the 0.5% level of significance.

### FW and SMD Resistance

Fourteen ILs in Trial 03 were resistant to FW, of which 12 were SMD resistant, while in Trial 04, 24 ILs were resistant to FW, of which 20 ILs were resistant to SMD. ILs combining resistance to FW and SD were further screened for resistance to these two diseases in the next season. The second-year evaluation confirmed SMD resistance in all ILs (12 ILs from Trial 03 and 20 ILs from Trial 04), whereas FW resistance was confirmed in 10 ILs (ICPIL # 17148, 17149, 17150, 17151, 17153, 17154, 17157, 17158, 17161, and 17162) from Trial 03 and 19 ILs (ICPIL # 17164, 17165, 17167, 17168, 17169, 17170, 17172, 17173, 17174, 17177, 17178, 17182, 17183, 17184, 17185, 17186, 17187, 17188, and 17191) from Trial 04.

### AMMI Analysis

The genotype, location, and genotype × location interactions (GEIs) were assessed by AMMI model in MET 01 (**Table S6a**) and MET 02 (**Table S7a**). Variance analysis of the AMMI model for grain yield showed significant effects for genotype, location, and GEI in MET 01 and MET 02. Locations contributed the largest phenotypic variation, followed by GEI and genotype in both MET 01 and MET 02 (**Tables S6a** and **S7a**). The GEI was highly significant (*P* ≤ 0.01), accounting for over 29% and 32% of the total variation in MET 01 and MET 02, implying the differential response of the genotypes to locations. The presence of GEI was also clearly demonstrated by the AMMI model when the interaction was partitioned into the first two interaction PC axes (IPCA) (**Tables S6a** and **S7a**). IPCA1 and IPCA2 scores were highly significant, explaining 48.2% and 34.8% of the variability, respectively, in MET 01 and 55.6% and 30.6% of the variability, respectively, in MET 02 (**Tables S6a** and **S7a**).

**Figure 1:**
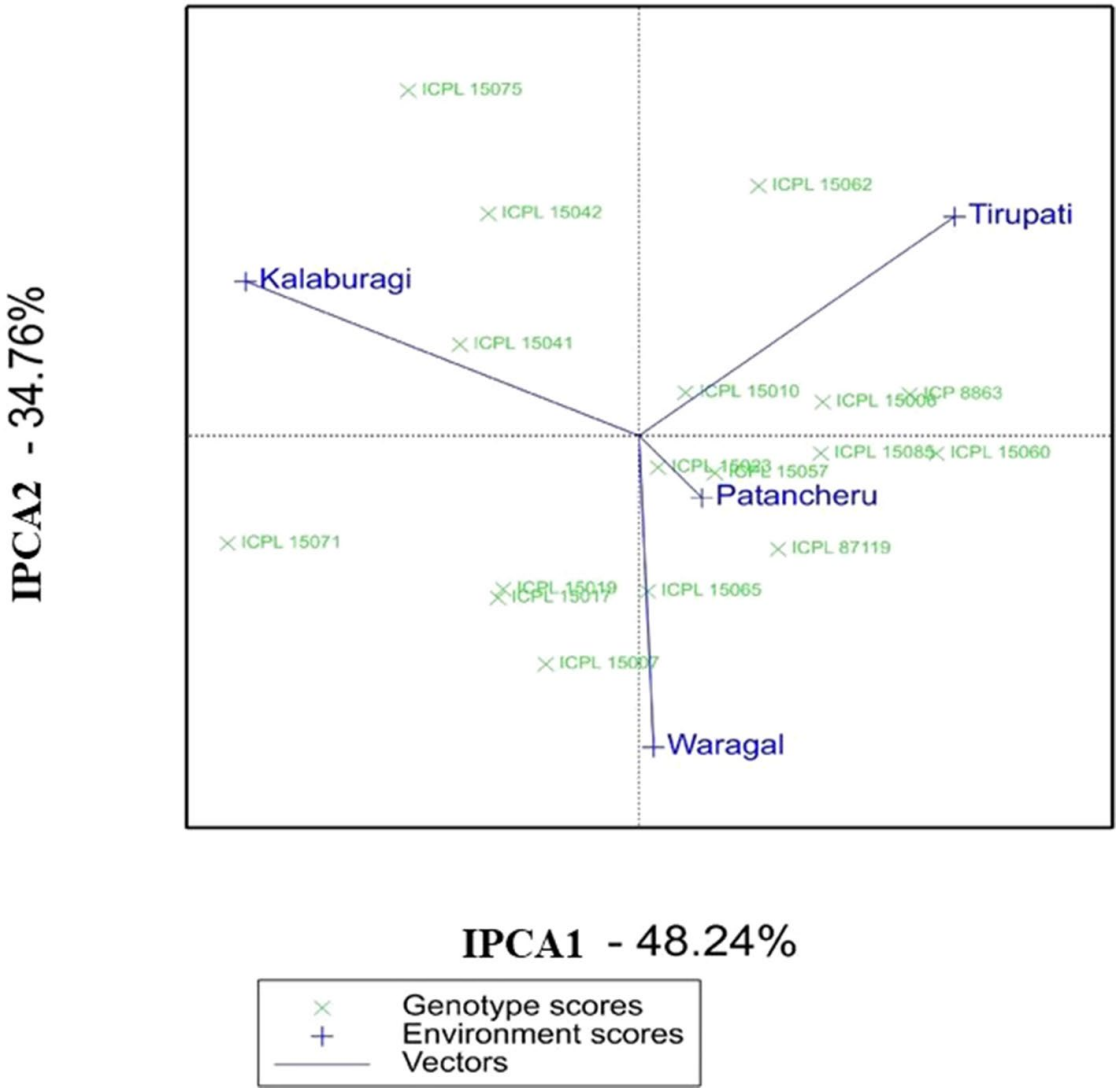
Additive main effects and multiplicative interaction (AMMI) biplot showing the first two principal axes of interaction (IPCA1 vs IPCA2) for the grain yield per plant of 15 introgression lines in MET 01 across four locations during the 2016 rainy season in India.

In the AMMI biplot [second interaction PC axis (IPCA2) against the first interaction PC axis (IPCA1)], genotypes closer to the origin of the axis have a smaller contribution to the interaction than those that are farthest. In the AMMI biplot for grain yield (**Figure 1**), ICPL 15023, ICPL 15010, and ICPL 15057 in MET 01 showed greater stability. Of these three ILs, the grain yield per plant of ICPL 15057 and ICPL 15010 was lower than the overall population mean and checks (ICP 8863 and ICPL 87119), whereas the grain yield of ICPL 15023 was better than the checks and population mean. From the AMMI biplot as well as AMMI selection per environment, it is evident that ICPL 15062, ICPL 15085, ICPL 15019, and ICPL 15075 were the best-suited ILs in Tirupati, Patancheru, Warangal, and Kalaburagi locations, respectively (**Figure 1** and **Table S6b**). Further, ICPL 15071 was better adapted at Kalaburagi and Warangal, whereas ICPL 15085 at Patancheru and Tirupati locations.

**Figure 2:**
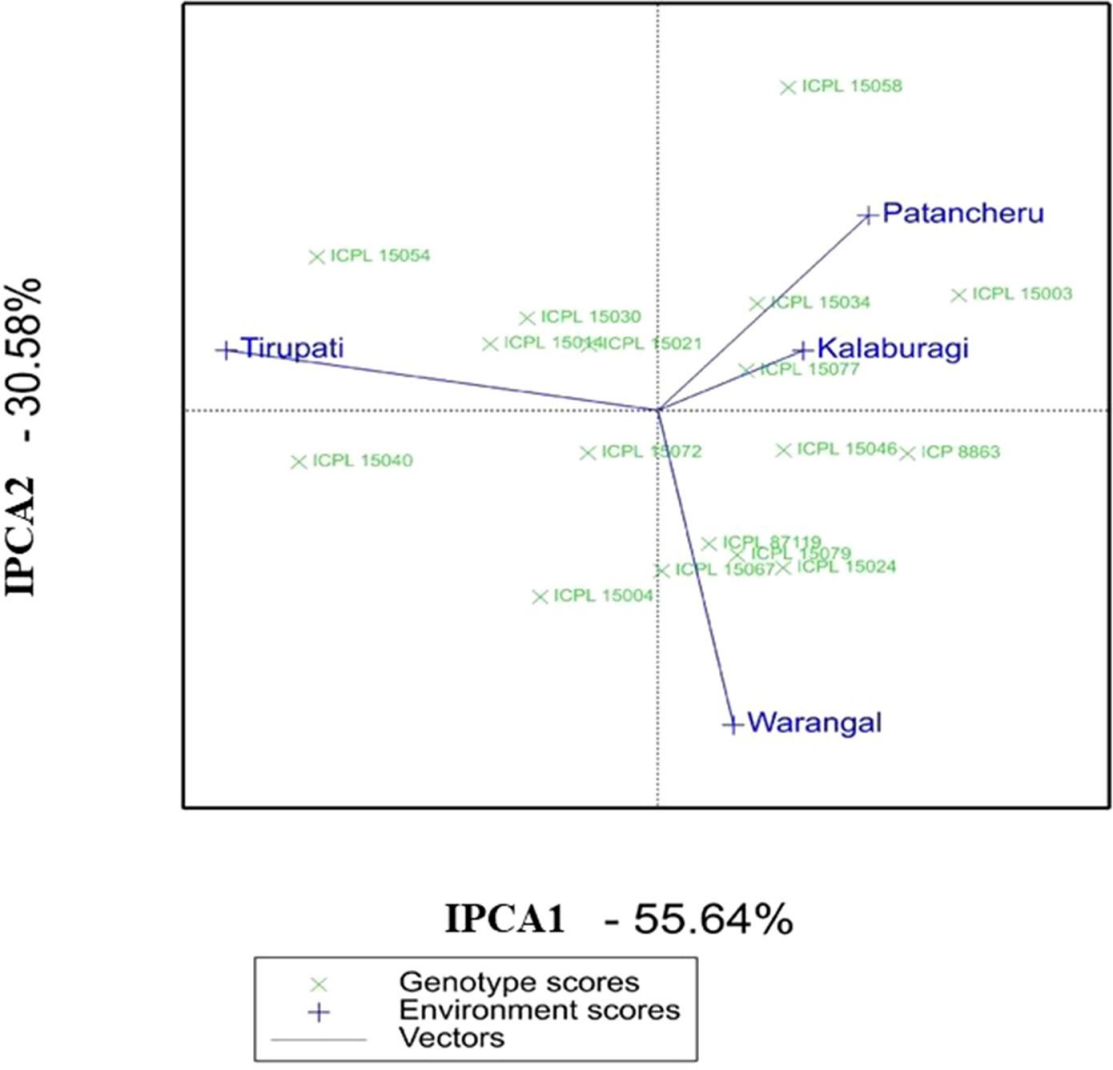
Additive main effects and multiplicative interaction (AMMI) biplot showing the first two principal axes of interaction (IPCA1 vs IPCA2) for the grain yield per plant of 15 introgression lines in MET 02 across four locations during the 2016 rainy season in India.

Similarly, in MET 02, the AMMI biplot (IPCA2 vs IPCA1) for grain yield per plant showed that ICPL 15072 was the most stable genotype across locations (**Figure 2**). ICPL 15077 and ICPL 15014 were found to be the best-suited ILs at Patancheru and Tirupati, respectively. ICPL 15077 was placed closer to both Kalaburagi and Patancheru environmental vectors and hence was suitable for these locations. Based on the AMMI selections per environment (**Table S7b**), this genotype was ranked number 1 at Patancheru and number 2 at Kalaburagi.

Apart from the AMMI biplot, AMMI stability analysis (ASV) gives the strength to quantify and classify genotypes that have stable performances across different environmental conditions (Oliveira et al., 2014). A low ASV of any genotypes indicates its stability across environments, while those with high ASV values are less stable. ICPL 15023, ICPL 15010, ICPL 15057, and ICPL 15065 were found to be the most stable ILs in MET 01 with ASV values of 0.4 to 1.5, whereas ICPL 15071 and ICPL 15075 were the most unstable ILs with ASV values of 5.0 and 4.3, respectively (**Table S6c**). In MET 02, ICPL 15072, ICPL 15021, ICPL 15077, and ICPL 15067 were the most stable lines based on ASV value (1.1–1.6) (**Table S7c**).

Stability with high yield potential should be considered for the selection, and hence, GSI may be useful in selecting the best genotypes. Based on low GSI value, ICPL 15023 and ICPL 15085 in MET 01 and ICPL 15072 and ICPL 15077 in MET 02 were found to be the most stable with high yield potential (**Tables S6c** and **S7c**).

## Discussion

Global warming is adversely impacting agricultural production globally. Developing climate-resilient crops and their cultivation will contribute to food and nutritional security to the growing world population. The narrow genetic base may result in the vulnerability of food crops and render them susceptible to stresses. Developing new climate-resilient cultivars necessitates the exploitation of new and diverse sources of variations in breeding programs. Crop wild relatives are the reservoir of many useful genes, and their use in breeding programs will lead to enhanced levels of plasticity in new cultivars and thereby a higher capability to withstand environmental stresses (Khoury et al., 2015; Sharma, 2017).

Though the potential of wild species in improving crop cultivars is well known, breeders are mostly indisposed to use these important and unexploited genetic resources in many breeding programs. Cross-incompatibility, poor adaptability, and linkage drag among others are the major constraints for low use of wild relatives in crop breeding. Moreover, difficulty in hybridization even with cross-compatible wild species and more time, efforts, and resources required to minimize linkage drag for the development of interspecific populations make the introgression breeding using wild relatives lengthier and cumbersome (Sharma et al., 2013). Pre-breeding provides a unique platform for creating new genetic variability following interspecific hybridization and developing ILs with preferred traits for genetic enhancement. Thus, ILs with higher frequency of useful traits introgressed from wild relatives provide new sources of variability into the diverse genetic background for use in breeding to develop climate-resilient crops (Sharma, 2017).

Use of wild species in breeding programs is often associated with introgressing many undesirable traits such as long maturity duration, pod shattering, and small pods, which are commonly known as linkage drag. Hence, for population development, an advanced backcross approach followed by selfing was used to recover the genetic background of the cultivated type and to identify promising recombinants with minimum linkage drag. To broaden the genetic base of pigeonpea, *C. acutifolius* (ICPW 12) and *C. cajanifolius* (ICPW 29) were crossed with recurrent parent ICPL 87119 (Asha), and the F_1_s were backcrossed twice and selfed for three to four generations to derive 75 ILs that were evaluated for stress tolerance and productivity to identify promising ILs with required characteristics that breeders may use to accelerate cultivar development in pigeonpea. The results showed that the advance backcross approach was successful in creating useful genetic variability with minimum linkage drag using wild species.

### ILs with Great Diversity in Phenology and Agronomic Traits

Large variation in maturity duration (141–176 days) as noted in the present study makes these ILs an ideal genetic resource for use in pigeonpea breeding worldwide. Pigeonpea cultivars based on maturity are categorized into super-early (<100 days), extra-early (100–120 days), early (120–140 days), mid-early (140–160 days), medium (160–180 days), and long-duration (>180 days) groups (Srivastava et al., 2012). Each maturity group is suited to a specific agro-ecosystem, which is defined by altitude, temperatures, latitude, and day length. India is a major pigeonpea-growing country, and a medium-duration variety, Asha (ICPL 87119), dominates the production for the past two decades (Kumar et al., 2014).

In the national system of India, more than 10-year-old varieties are not promoted in the seed chain and are termed as “extant” varieties. As Asha was released in 1995 for cultivation, there is no possibility to promote this variety in the seed chain. Hence, there is a dire need to introduce new high-yielding varieties with FW and SMD resistance as a replacement to Asha. The high-yielding ILs such as ICPL 15085, ICPL 15072, ICPL 15062, ICPL 15067, ICPIL 17164, ICPIL 17165, and ICPIL 17169 identified in the present study, having on and average 21–50% yield superiority over Asha and with average maturity ranging from 161 to 170 days across locations and/or over years, provide a great opportunity for breeders using this useful genetic resource to develop new cultivars that may replace Asha.

Further, due to short cropping seasons, pigeonpea improvement programs are focusing on developing short-duration varieties, particularly in the mid-early maturity duration group. ICPL 15010, ICPL 15019, ICPL 15023, ICPL 15021, ICPL 15077, and ICPIL 17160 across locations and/or years were more high yielding and matured earlier (<160 days) than Asha and hold great potential in developing high-yielding varieties in the mid-early maturity duration group.

Further, based on the mean phenotypic diversity index, the most diverse pairs of ILs have been identified. It will be interesting and fruitful to involve the most diverse ILs in hybridization programs to see the extent of segregations for different traits. Besides this, a few promising ILs such as ICPL 15065, ICPL 15014, and ICPIL 17167 were found to be more diverse than the recurrent parent ICPL 87119. Thus, these lines may be used to broaden the genetic base of cultivated pigeonpea.

### Yield Stability

The AMMI, based on the two-way ANOVA and the PCA, is a unified approach to analyze multilocation trial data (Crossa et al., 1990). Being a powerful tool for visualizing as well as partitioning the GEI, AMMI determines the stable genotypes and the behavior of test environments (Silveira et al., 2013). A large SS for the environment in AMMI analysis showed that the environments in which these lines were evaluated were highly diverse. ICPL 15023 in MET 01 and ICPL 15072 in MET 02, being close to the AMMI biplot origin, are the most stable ILs across environments. These lines may be further evaluated on large-scale trials prior to recommending for cultivation. These two lines also scored high based on grain yield, ASV, and GSI. Hence, these lines should be given utmost importance for use in breeding programs or release them directly as a variety. The AMMI biplot further revealed that ICPL 15058, ICPL 15075, and ICPL 15071 are adapted to specific environments and therefore may be used in breeding for developing region-specific cultivars or may be deployed as a cultivar for production in specific environments.

### Biotic Stress Resistance

SMD and FW cause substantial losses to pigeonpea production and have been identified as the “must-have” traits for pigeonpea in India. In this study, the majority of the lines in Trial 03 and Trial 04 were resistant to either FW, SMD, or both. Twenty-nine ILs from both the trials showed high levels of resistance (<10% incidence) for both SMD and FW. *C. acutifolius* and *C. cajanifolius* were reported resistant to SMD (Khoury et al., 2015; Patil and Kumar, 2015). Ten C. *acutifolius*-derived ILs and 11 *C. cajanifolius*-derived ILs showed complete resistance to SMD (0% incidence), implying that SMD resistance has been successfully introgressed into these lines.

Three distinct isolates have been characterized for SMD, namely, Bangalore, Patancheru, and Coimbatore isolates; the Patancheru and Coimbatore isolates are mild strains, while the Bangalore isolate is the most virulent one (Kulkarni et al., 2003). A breakdown of SMD resistance has been reported based on multilocation field trials (Nene et al., 1989). The SMD resistance sources identified in the present study should be screened further across locations to identify isolate-specific sources of SMD resistance.

### ILs: Potential To Be Released as Cultivars in India

The superiority of a few ILs over local and/or national checks provided an opportunity to the breeders to include a few promising ILs in the initial varietal trials (IVTs) of the All India Coordinated Research Project (AICRP) on pigeonpea for a wider evaluation across different agro-ecological zones in India. ICPL 15010, ICPL 15072, and ICPL 15062 based on their high yield compared to local checks and market preference for seed color and size have been nominated for IVT of the AICRP on pigeonpea under mid-early and medium maturity duration categories. ICPL 15010 has been nominated under the mid-early maturity duration group, whereas ICPL 15072 and ICPL 15062 are under the medium-maturity duration group. Besides India, Myanmar is the second-highest pigeonpea-producing country and is dominated by a long-duration variety, Monywa Shwedingar. These promising lines have also been shared with researchers in Myanmar for use in pigeonpea breeding programs. Utilization of these promising ILs derived from wild *Cajanus* species in pigeonpea breeding programs will assist in developing new climate-resilient cultivars with a broad genetic base.

## Data Availability Statement

All datasets generated for this study are included in the articles/**Supplementary Files**.

## Author Contributions

SS conceived the idea; SS and CK coordinated the project; SS was involved in developing the pre-breeding populations; SS and CK selected material for this study and constituted the trials; CK, PR, LP, and SM evaluated the material across locations; MS screened the material for FW and SMD; PP assisted in statistical data analysis; SS and PP prepared the manuscript; CK, PR, LP, SM, and MS provided their inputs. All the authors reviewed and approved the final manuscript.

## Funding

Funding support provided by the Global Crop Diversity Trust (GCDT), (Grant Number GS15020), and CGIAR Research Program on Grain Legumes and Dryland Cereals (GLDC).

## Conflict of Interest

The authors declare that the research was conducted in the absence of any commercial or financial relationships that could be construed as a potential conflict of interest.
